# Behavioral or neuropsychiatric symptoms of Alzheimer's disease: from psychopathology to pharmacological management

**DOI:** 10.1055/s-0043-1777774

**Published:** 2023-12-29

**Authors:** Antonio Lucio Teixeira, Natalia Pessoa Rocha, Jennifer Gatchel

**Affiliations:** 1 University of Texas University of Texas Health Science Center, McGovern Medical School, Department of Psychiatry and Behavioral Sciences, Neuropsychiatry Program, Houston, Texas, United States.; 2 Faculdade Santa Casa Belo Horizonte, Belo Horizonte MG, Brazil.; 3 University of Texas Health Science Center, McGovern Medical School, Department of Neurology, Houston, Texas, United States.; 4 Massachusetts General Hospital, Department of Psychiatry, Boston, Massachusetts, United States.; 5 Baylor College of Medicine, Department of Psychiatry, Houston, Texas, United States.; 6 Michael E. Debakey VA Medical Center, Houston, Texas, United States.

**Keywords:** Alzheimer Disease, Neuropsychiatry, Apathy, Depression, Anxiety, Psychomotor Agitation, Psychotic Disorders, Sleep, Doença de Alzheimer, Neuropsiquiatria, Apatia, Depressão, Ansiedade, Agitação Psicomotora, Transtornos Psicóticos, Sono

## Abstract

Neuropsychiatric or behavioral symptoms of dementia encompass a series of disorders, such as anxiety, depression, apathy, psychosis, and agitation, all commonly present in individuals living with dementia. While they are not required for the diagnosis of Alzheimer's disease (AD), they are ubiquitously present in all stages of the disease, contributing to negative clinical outcomes, including cognitive decline, functional disability, and caregiver burden. Neuropsychiatric symptoms have been conceptualized not only as risk factors but as clinical markers of decline along the AD spectrum. The concept of “mild behavioral impairment”, the behavioral correlate of mild cognitive impairment, has been proposed within this framework. The first steps in the management of behavioral symptoms in AD involve defining the target and investigating potential causes and/or aggravating factors. Once these factors are addressed, non-pharmacological approaches are preferred as first-line interventions. Following the optimization of anticholinesterase treatments, specific pharmacological approaches (e.g., antidepressants, antipsychotics) can be considered weighing potential side effects.

## Resumo

Sintomas neuropsiquiátricos ou comportamentais de demência envolvem uma série de condições, como ansiedade, depressão, apatia, psicose e agitação, frequentemente observadas em indivíduos com demência. Embora esses sintomas não sejam necessários para o diagnóstico da doença de Alzheimer, estão presentes em todas as fases ou estágios da doença, contribuindo negativamente para o declínio cognitivo, comprometimento funcional e sobrecarga do cuidador. Os sintomas neuropsiquiátricos têm sido conceituados não apenas como fatores de risco, mas também como marcadores clínicos de progressão da doença de Alzheimer. O construto “comprometimento comportamental leve”, correlato comportamental do comprometimento cognitive leve, tem sido proposto nesse contexto. Os primeiros passos na abordagem dos sintomas comportamentais da doença de Alzheimer envolvem definir os alvos-terapêuticos e investigar potenciais causas ou fatores agravantes. Após intervir nesses fatores, abordagens não farmacológicas constituem a primeira linha de intervenção. Depois da otimização do tratamento anticolinesterásico, terapias farmacológicas específicas (por exemplo, antidepressivos, antipsicóticos) podem ser consideradas, levando-se em conta potencias efeitos colaterais.

## INTRODUCTION

**Figure 1:**
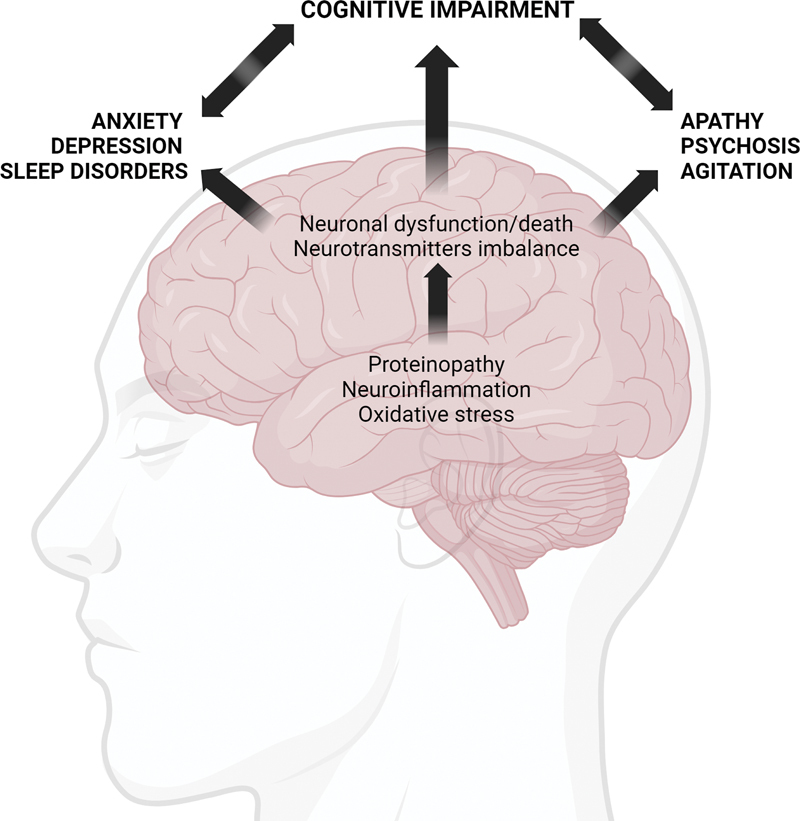
AD-related pathophysiological mechanisms potentially link cognitive impairment and behavioral symptoms. Proteinopathy (abnormal accumulation of amyloid-β and tau protein), neuroinflammation, and oxidative stress contribute to neuronal dysfunction and, ultimately, neuronal death in AD. The consequent neurotransmitter imbalance and neural circuit dysfunction result in cognitive impairment and behavioral symptoms. Cognitive and behavioral symptoms tend to aggravate each other.

Alzheimer's disease (AD) is the leading cause of dementia, a clinical syndrome characterized by a decline in cognitive functions with functional repercussions. 1 Behavioral symptoms are not required for diagnosis, but they are frequently recognized in AD, especially in its moderate to severe stages. 2 Actually, they may occur in all stages of AD, including preclinical and prodromal stages, and virtually all patients will develop at least one behavioral disorder during the course of the disease. 3 Cross-sectional studies have shown variable prevalence numbers, with rates varying according to sample characteristics, measurement tools, and study setting, with higher rates observed in institutionalized patients. 4 The pathophysiological mechanisms underlying AD presentation overlap with those associated with neuropsychiatric syndromes, and the relationship between cognitive and behavioral symptoms is thought to be bidirectional ( Figure 1 ).

Behavioral symptoms vary not only according to the stage of neurodegeneration but also the underlying disease. 5 6 For instance, psychosis can affect almost three-quarters of patients with dementia with Lewy bodies, while it is less frequent in AD. Behavioral problems are core features of non-AD types of dementia and usually guide their diagnostic process. 5 6 Identifying vivid visual hallucinations strongly suggests Lewy body disease, while inappropriate social behavior may indicate a behavioral variant of frontotemporal dementia.

The literature usually refers to behavioral symptoms as behavioral and psychological symptoms of dementia (BPSD) or neuropsychiatric symptoms of dementia (NPS). 6 7 These are ‘umbrella’ terms that encompass a series of symptoms, such as agitation, anxiety, apathy, depression, delusion, and hallucination, among others.

Auguste D., the patient originally described by Alois Alzheimer, had more pronounced behavioral problems (related to delusions and hallucinations) than cognitive ones. 8 In many cases, behavioral symptoms can indeed be more troublesome for patients and caregivers than impaired cognition and the related functional decline. Moreover, behavioral symptoms negatively influence AD-related outcomes, including cognitive decline, functional disability, caregiver burden, costs, medical service utilization, and risk of institutionalization and death. 7 9 10

Despite their clinical relevance, their management remains challenging, and until very recently, there was no single FDA-approved pharmacological therapy for behavioral symptoms in dementia. The present narrative review will provide an updated perspective of the field, discussing current trends in the classification and treatment of different behaviors along with emerging topics, such as mild behavioral impairment.

## MILD BEHAVIORAL IMPAIRMENT

While cognitive symptoms remain the core clinical feature of AD, behavioral symptoms are very common. Behavioral or neuropsychiatric symptoms can occur at any disease stage, and they often manifest very early in the AD continuum, i.e., since its prodromal stages. Their importance was emphasized by the 2011 National Institute on Aging–Alzheimer's Association (NIA-AA) revised guidelines for dementia diagnosis, wherein the 1984 core criteria were modified to explicitly mention “ *changes in personality, behavior, or comportment—symptoms include uncharacteristic mood fluctuations such as agitation, impaired motivation, initiative, apathy, loss of drive, social withdrawal, decreased interest in previous activities, loss of empathy, compulsive or obsessive behaviors, socially unacceptable behaviors* ” 11 .

Behavioral syndromes are also frequently seen as risk factors for AD, increasing the incidence of dementia in both individuals with mild cognitive impairment (MCI) and cognitively normal adults. 12 13 As acknowledged in the aforementioned criteria, behavioral symptoms have been conceptualized not only as risk factors but as clinical markers of cognitive and functional decline along the AD spectrum. Assuming that neurodegeneration can manifest as behavioral or neuropsychiatric symptoms even before any clinically defined cognitive impairment, Dr. Taragano et al. proposed the mild behavioral impairment (MBI) construct. 14 15 16 Recently, the International Society to Advance Alzheimer's Research and Treatment (ISTAART) defined MBI as a broad concept that describes a syndrome comprising sustained and impactful late-life neuropsychiatric symptoms (emerging after 50 years of age) that represent a clear change from an individual's baseline and can be early manifestations of neurodegenerative disease, 13 occurring in cognitively unimpaired individuals or in those with MCI. In other words, MBI can be understood as the behavioral correlate of MCI in the spectrum of neurodegenerative diseases.

MBI comprises five domains: motivation, affective regulation, impulse control, social cognition, and perception/thought content. 13 For MBI, behavioral symptoms can be of any severity, should precede or occur concurrently with cognitive impairment, and persist for at least six months. By definition, symptoms occurring after the onset of dementia cannot be MBI. 13 17 The NPS Professional Interest Area of ISTAART developed a rating scale to operationalize MBI. The MBI checklist (MBI-C) is a 34-item instrument that describes and measures changes in a selected list of behavioral symptoms that reflect the five MBI domains of decreased motivation, emotional dysregulation, impulse dyscontrol, social inappropriateness, and abnormal perception or thought content. The MBI-C is a tool intended for use in clinical and research settings to detect and measure changes in NPS over time. 17

MBI has been proposed as a diagnostic category capable of identifying patients at increased risk of developing dementia regardless of the presence of cognitive symptoms. It may help predict the risk of several dementias, including but not exclusive to AD. 17 The main rationale for using MBI criteria is the potential to detect neurodegeneration at early stages when therapeutic interventions would be more likely to change disease progression. The pathophysiological processes underlying AD and related dementia start decades before their clinical diagnoses, and although preclinical AD has been recognized as one disease stage in the revised NIA-AA criteria for AD diagnosis, 11 18 so far, there is no test to predict if someone will develop clinically defined AD. Accordingly, the identification of people with MBI will help define an ‘at high risk’ group for dementia, which is an important step towards the development of effective disease-modifying therapies. Moreover, the treatment of the related behavioral symptoms (as early manifestations of AD) may slow the progression or mitigate the presentation of dementia. 13 As a word of caution, the diagnostic criteria for MBI represent a significant advance in the definition of behavioral presentations of AD and related dementias, but the concept of MBI still needs to be validated from biological and clinical standpoints. 13

## ASSESSMENT AND MANAGEMENT OF BEHAVIORAL SYMPTOMS IN AD: GENERAL PRINCIPLES

For the assessment of behavioral symptoms in AD, the most used tool is the Neuropsychiatric Inventory (NPI). 5 Different versions of the NPI can be applied according to the goal or context. In research, the NPI-C, or clinician-version, is frequently used. The clinician scores the behaviors based on the direct assessment of the patient's behavior and taking into account the caregiver and patient's reports. It is more time-consuming but may be less biased than the NPI-Q, which relies only on the partner's report. On the other hand, the NPI-Q is brief and convenient (e.g., can be filled out by the care partner before the appointment), providing an overview of the behaviors presented by the patient and the related impact. Therefore, the NPI-Q may help a busy clinician in their clinical practice. 2 Of note, there are other general tools, such as the Behavioral Pathology in Alzheimer's Disease Rating Scale (BEHAVE-AD), 19 and dedicated instruments for specific behaviors, such as the Cornell Scale for Depression in Dementia, 20 but they are mainly used in research.

The NPI assesses 12 distinct behaviors that cover the most clinically relevant issues ( Table 1 ). In the past, partly because of the overemphasis on cognitive features of AD, these behaviors were lumped together under umbrella terms like BPSD and NPS and not considered individually. 21 22 Conversely, many of these behaviors actually overlap in clinical practice. Recognizing this overlap, several studies applied statistical methods (e.g., principal component analysis, classic factor analysis, latent class analysis) to reduce the dimensions of behavioral symptoms in AD. 23 While there were discrepant results, most studies proposed three to four domains or dimensions that could explain behavioral variability: affective (including apathy, anxiety, depression/dysphoria), psychosis (delusion, hallucination), agitation (aberrant motor behavior, aggression), and disinhibited (elation, disinhibition). These dimensions are clinically relevant as they can guide management and therapeutic interventions. 21

**Table 1:** Behavioral symptoms of Alzheimer's disease

Behavioral symptom	Features
Agitation	Pacing, restlessness, repetitive motor and vocal behaviors
Aggression	Verbal: screaming, cursing Physical: hitting, pushing, scratching, biting, spitting, destroying objects or property
Anxiety	Tense, worried
Apathy	Indifferent, reduced goal-directed behaviors, socially withdrawn, blunted emotional response
Depression	Sadness, tearful, anhedonia, negative thoughts (hopelessness, guilt, death)
Disinhibition	Impulsivity, socially inappropriate behaviors such as tactless or rude remarks, sexual comments, exposure/undressing, masturbation
Elation/Euphoria	Feeling excessively well or happy
Irritability	Impatience, increased reactivity
Psychosis	Delusions: paranoid/persecutory, misidentification, schizophreniform (e.g., thought insertion, broadcasting)
	Hallucinations: visual, auditory, somatic
Eating behaviors	Anorexia, hyperphagia, eating inappropriate substances (pica)
Sleep disorders	Insomnia, hypersomnia, restless leg, REM-related behavior, sleep-disordered breathing (e.g., obstructive sleep apnea)

The first step in managing behavioral symptoms in AD is to define the presence and severity of each disruptive behavior. With a defined goal, the second step is to investigate potential causes and/or aggravating factors. These factors can be categorized as patient-, caregiver-, and environment-related 22 ( Table 2 ). Once these factors are addressed (e.g., urinary tract infection treatment, environmental stimuli control), the next stage is to plan the therapeutic interventions. There is a consensus that non-pharmacological approaches are preferred as first-line interventions. 24 Discussing these approaches is beyond the scope of this text, but it is important to highlight that many have been evaluated in controlled trials with positive results. 25 Besides that, these interventions are much safer than pharmacological therapies. A major limitation of non-pharmacological interventions is their access, which may depend on multiple factors, such as the availability of caregivers and/or therapists, and financial resources.

**Table 2:** Individual-, caregiver- and environment-related factors implicated in the development and/or persistence of behavioral symptoms in Alzheimer's disease

Individual	Caregiver	Environment
Sensory deprivation (e.g., impaired vision, hearing)	Limited training or experience	Changes in the environment
Sensory overstimulation	Distress and related mental issues	Lack of a structured routine
Physical discomfort (e.g., pain, hunger, thirsty)	Constant changes	Excessive or lack of stimulation
Medical comorbidity (e.g., urinary tract infection)		Uncomfortable (e.g., hot, cold) and/or unsafe place

Regarding pharmacological approaches, some general principles must be followed: starting at a low dose, slowly titrating up to the minimum effective dose, and periodically assessing side effects and the need for continued medication prescription. 26 This is very important because, in contrast to the progressive cognitive decline that defines AD, behavioral symptoms fluctuate during the course of the disease. 27 28 The decision on the specific medication must consider the targeted behavioral symptom or domain, the patient's medical comorbidities, and polypharmacy. 29 30 For instance, for affective symptoms, antidepressants, mainly selective serotonin reuptake inhibitors (SSRIs), are the primary choice. Optimization of anticholinesterase treatment seems to contribute to these symptoms as well. Emerging evidence has indicated that this class of medication is helpful not only in managing cognitive and other behavioral symptoms but also positively affects dementia-related outcomes (i.e., risk of death or severe dementia). 31 32 33 For agitation, antidepressants can also be tried, while atypical antipsychotics should be reserved for severe cases and used after weighing the associated cardiovascular and death risks (see below).

The contemporary trend is to define a Diagnostic and Statistical Manual (DSM)-like set of criteria for specific behaviors, such as apathy, agitation, and psychosis, as it will be discussed in the following sections.

## APATHY, DEPRESSION, AND ANXIETY

Apathy is a behavioral syndrome defined by reduced or loss of motivation for goal-directed behaviors and cognitive activities alongside blunted aﬀect. 34 Apathy is the most common behavioral disorder in AD, with a prevalence of up to 90% in severe dementia. It has been associated with a series of negative clinical outcomes, including cognitive and functional decline, caregiver burden, institutionalization, and mortality, explaining a significant portion of the impact of behavioral or neuropsychiatric symptoms in AD. 34 Among all behavioral symptoms, apathy is the one closer correlated with cognitive decline in AD, predicting progression from normal cognition to MCI and from MCI to dementia. 35

The International Society for CNS Clinical Trials and Methodology and the European Psychiatric Association proposed diagnostic criteria for apathy in neurocognitive disorders within a framework similar to the DSM. Accordingly, an individual with AD is diagnosed as apathetic when they meet four criteria (A-D). 36 Criterion A requires the diagnosis of cognitive impairment or dementia. Criterion B requires the presence of at least one persistent or recurrent symptom in at least two of three domains (i.e., diminished initiative, diminished interest, diminished emotional expression/responsiveness) for a minimum of four weeks. For example, a patient may be less likely to spontaneously initiate usual activities, such as hobbies, less interested in participating in everyday activities, and emotionally blunted. Criterion C is exclusionary and postulates that these symptoms are not solely explained by physical or motor disabilities, impaired arousal, the effect of a substance, or a psychiatric disorder. Criterion D states that these symptoms cause significant impairment in personal, social, occupational, and other areas of functioning.

Apathy has been conceptualized as a transdiagnostic syndrome within the disorders of motivation and reward. 34 37 Dopaminergic circuits play a major role in this context, thus pharmacological approaches activating dopamine signaling have been used for apathy. Methylphenidate was shown to be an eﬀective treatment for apathy in AD in open and controlled studies but with concerns related to potential weight loss, increased anxiety, and cardiovascular effects. 38 39

There is considerable overlap (30-50%) between apathy and depression in AD. 34 40 Depression is also a very common behavioral problem in AD, with an estimated prevalence of major depression in AD around 15%, with higher numbers when milder forms of depressive disorders are considered. 41 As the lack of motivation is one of the DSM criteria for the diagnosis of major depression, the differential diagnosis between apathy and depression can be challenging. 40 In clinical practice, depression can be diﬀerentiated from apathy as the former involves subjective feelings of sadness, negative thoughts (e.g., guilt, hopelessness, low self-esteem), and sleep disorders (e.g., early awakening).

There are National Institute of Mental Health (NIMH)-sponsored diagnostic criteria for depression in AD based on the DSM criteria for major depression. 42 The criteria for depression in AD require the presence of three – at least one must be depressed mood or decreased positive affect/pleasure in response to usual activities – out of ten symptoms (depressed mood, decreased positive affect or pleasure, social isolation, disruption in appetite, disruption in sleep, psychomotor changes, irritability, fatigue, recurrent thoughts of death or suicide, and feelings of worthlessness, hopelessness or guilt) during a two-week period, with a definite change from the previous level of functioning. In addition to the NPI, the Cornell Scale for Depression in Dementia has been used to assess depression in AD. 20 As it is a clinician-rated scale considering both patient and caregiver perspectives, the Cornell Scale for Depression in Dementia can better control biased information related to anosognosia, normalization of symptoms, and/or caregiver stress. 43 Pharmacological therapy for depression in AD usually relies on SSRIs (e.g., sertraline, citalopram), 40 with a growing tendency to use dual antidepressants, such as duloxetine, venlafaxine/desvenlafaxine. 43 It is worth mentioning that antidepressants may worsen apathy. 34

While apathy and depression have been relatively well investigated in AD, much less is known about anxiety. 44 Anxiety is the third most common behavioral disorder in AD, with an estimated prevalence of around 40%. 4 In the early stages of AD, anxiety resembles its presentation in adults, i.e., characterized by worries and somatic symptoms, while in later phases of the disease, it manifests as emotional distress, irritability, and excessive motor activity. Moreover, anxiety tends to aggregate with depression within an affective behavioral domain but is less associated with cognitive decline in AD than depression and apathy. The management of anxiety in AD emphasizes non-pharmacological strategies (e.g., physical activity, music, occupational and cognitive therapies), especially given the significant risks of using benzodiazepines in older adults, such as cognitive decline, falls, and sedation. SSRIs are the drugs of choice for anxiety in AD, and trazodone can be considered when associated with insomnia. 44

## PSYCHOSIS

Psychosis is a descriptive term encompassing delusions and hallucinations. 45 46 Psychosis is a clinically relevant neuropsychiatric syndrome reported in 41% of patients with AD, with delusions in 36% and hallucinations in 18%. 47

From a psychopathological perspective, there are different types of delusions (e.g., persecutory, religiosity, misidentification) and hallucinations (e.g., visual, auditory, somatic) in AD. Furthermore, they may differ during the course of the disease. Paranoid or persecutory delusions, including delusions of theft or infidelity, tend to occur earlier than misidentification delusions. 48 The latter delusions are commonly associated with more severe cognitive impairments, mainly perceptual deficits (e.g., prosopagnosia), and include Capgras (or hypofamiliarity), in which the patient firmly believes that a relative or a friend is an impostor, Fregoli (or hyperfamiliarity), convinced that a close person constantly changes their appearance or incarnate in others, phantom boarder syndromes, the belief that uninvited people are living in the house, and reduplicative paramnesias, the belief that a place a familiar place exists in two or more physical locations simultaneously. 48 Hallucinations often present with delusions rather than alone, frequently overlapping with them, such as misidentification syndromes. Somatic hallucinations are also difficult to disentangle from somatic delusions, as in Ekbom syndrome or delusional parasitosis. 49 Visual hallucinations are the most frequent, and hallucinatory phenomena can range from simple (e.g., unformed images, noises) to complex characteristics (e.g., people, conversations).

Psychosis tends to persist for months, 47 and this protracted course helps to discriminate it from delirium, which usually has an acute or subacute presentation. It is also important to distinguish delusions from confabulations, i.e., the fabrication of false or distorted memories. Psychosis can be distressing to the patient, leading to agitation and/or aggression, especially when confronted by a caregiver or a family member. Acknowledging these features is important to establish a non-pharmacological approach that includes a non-confrontational or argumentative attitude while attempting to ensure the patient's safety.

Antipsychotics have a modest effect on the treatment of psychotic symptoms in AD. Still, this effect comes at the cost of potentially harmful side effects, including cognitive, motor, and cardiovascular events and increased risk of death. 50 51 52 53 As typical antipsychotics (e.g., haloperidol, chlorpromazine) are associated with higher rates of side effects compared to atypical ones, most of the literature has focused on the latter. Meta-analytical evidence of atypical antipsychotics has shown small but statistically significant benefits with aripiprazole, olanzapine, and risperidone compared to quetiapine. 54 A network meta-analysis found that aripiprazole was the most effective and safe atypical antipsychotic for behavioral symptoms – as assessed by total scores in general psychopathology tools, not only psychosis domains – olanzapine provided the least benefit overall, while risperidone and quetiapine had intermediate profiles of effectiveness and safety. 55 However, a more recent meta-analysis of atypical antipsychotics (quetiapine, risperidone, olanzapine, aripiprazole, and brexpiprazole) for the treatment of AD-related psychosis failed to show any statistically significant improvement in symptoms, but all were associated with greater odds of mortality. 56 Despite their risk and effectiveness controversies, approximately one-third of patients with dementia use antipsychotics, with marked variation according to care settings and severity of disease. 57 Community-based studies have shown a much lower frequency of antipsychotic use when compared with long-term care settings (12.3% vs. 37.5%), while the prevalence of antipsychotic use increases from 6.7% in mild to 12.2% in moderate and up to 45.1% in severe dementia. 57

In this scenario, alternative pharmacological strategies for psychosis have been tested. Pimavanserin is a highly selective serotonin 5-HT2A receptor inverse agonist and antagonist with minimal dopaminergic, histaminergic, and muscarinic effects. This unique profile drug has shown to be effective for psychosis in Parkinson's disease, being FDA-approved for this indication. 58 Tariot et al. 59 conducted a phase 3, double-blind, randomized, placebo-controlled discontinuation trial with patients with psychosis related to AD and other non-AD dementias. Patients received open-label pimavanserin for 12 weeks. Those with a reduction from baseline of at least 30% in psychotic symptoms were randomly assigned at week 12 in a 1:1 ratio to continue receiving pimavanserin or to receive a placebo for up to 26 weeks. A relapse occurred in 12 of 95 patients (13%) in the pimavanserin group and in 28 of 99 (28%) in the placebo group (hazard ratio, 0.35; P = 0.005), favoring the former. Side effects occurred in 41% of the pimavanserin group and included headache, constipation, urinary tract infection, and asymptomatic QT interval prolongation. Despite promising efficacy results and an acceptable side-effect profile, the study was underpowered to show significant effects in specific types of dementia. In our experience, pimavanserin can be used in AD patients with less severe psychosis, but it should not be used in association with atypical antipsychotics because of safety concerns. 60

Finally, it is worth highlighting marked advances in the understanding of the neurobiology of psychosis in AD, as reviewed elsewhere. 61 Besides the overlap between the genetic architecture of AD-related psychosis and primary psychiatric disorders presenting with psychosis (schizophrenia, bipolar disorder), reinforcing the transdiagnostic validity of psychosis, studies have shown the contribution of Lewy body and vascular pathology.

## AGITATION

Agitation is a very troublesome behavioral problem in AD with major impacts, including costs and caregiver burden. 62 63 In a community-dwelling study involving older adults with a range of cognitive impairments, the prevalence of agitation ranged from 8.3% to 48.9%. 64 Agitation increases with dementia severity, and even higher rates (> 50%) are observed in nursing homes. 65

The International Psychogeriatric Association consensus panel considered not only excessive motor activity (e.g., restlessness, pacing, repetitive mannerisms) but also verbal (e.g., yelling, using profanities) and physical (e.g., hitting, scratching, biting, throwing objects) aggression under the definition of agitation. 62 Although agitation is not a synonym for aggression, the panel acknowledged that most studies do not discriminate against these behaviors and that this position also reflects how clinicians and families address the problem. According to the same criteria, these behaviors should be intermittently or persistently present for at least two weeks, which excludes acute agitation. Acute agitation must prompt careful investigation of environmental (e.g., movement to an unfamiliar palace) and medical causes (e.g., physical distress, pain, medication-related, urinary tract infection), ruling out agitated delirium.

Two different types of aggression – reactive and proactive – have been recognized. 62 The former seems to be more frequent in the context of dementia, while the second is mainly associated with psychotic phenomena. In line with this difference, it is worth noticing that agitation and psychosis usually do not load on the same behavioral domains (see above: Assessment and management of behavioral symptoms of AD). The relationship between agitation and other behavioral symptoms, such as affective and disinhibition/impulsive, tends to follow the same pattern, as they tend to load in discrete domains. 23 Another important clinical aspect is the definition of whether agitation has a circadian rhythm, such as in ‘sundowning’ and nighttime disruptive behaviors.

Recently, the International Psychogeriatrics (IPA) Agitation Work Group proposed an algorithm for managing agitation in neurocognitive disorders. 63 Using the ‘Investigate, Plan, and Act’ (also IPA) approach, in which the investigation of potential causative or contributor factors is followed by planning and acting, with reiterations of the process until agitation is controlled, the Working Group emphasized the integration of psychosocial and pharmacologic interventions. Psychosocial interventions that have multiple components target the caregiver (e.g., communication skills, education, self-care) and the patient (e.g., person-centered, aerobic activities, music, massage) and are delivered with regular follow-up have more therapeutic benefit. 66 67 Pharmacological treatment should be considered only after psychosocial interventions failed to reduce agitation. Before adding any new medication, it is fundamental to review the current regimen in search of drugs that can cause or aggravate agitation. For instance, benzodiazepines, opiates and anticholinergic drugs can cause delirium. Dopamine-blocking agents can cause akathisia, a condition characterized by subjective and objective restlessness and typically seen in patients taking antipsychotics. 68

For nighttime disruptive behaviors, trazodone and orexin receptor antagonists (e.g., suvorexant, lemborexant) have been used [63]. Of note, a recent double-blind, placebo-controlled study (SYMBAD) found no effect of mirtazapine – an atypical antidepressant with sedative properties at lower doses but activating in higher – on AD-related agitation and potentially increased mortality associated with its use. 69 Actually, antidepressants can be used as the first-line pharmacological strategy given the emotional distress usually underlying agitation. Citalopram is the most well-studied antidepressant and has been shown to reduce agitation in AD, especially those with mild to moderate agitation and less cognitive impairment. 70 71 As discussed in the previous section, atypical antipsychotics should be reserved for more severe cases. Risperidone is approved for agitation in dementia in some countries (e.g., Australia, Canada, United Kingdom), and brexpiprazole was recently approved in the United States. Actually, brexpiprazole was the first Food Drug Administration (FDA)-approved drug for an AD-related behavioral symptom, i.e., agitation. 72 The FDA granted a ‘Fast Track’ designation – that addresses a serious condition and fills an unmet medical need – to the application consisting of two 12-week, randomized, double-blind, placebo-controlled, fixed-dose studies. In the first study, patients received 1 or 2 mg, while patients received 2 or 3 mg in the second study, with the primary efficacy endpoint being the change from baseline in the Cohen-Mansfield Agitation Inventory total score at week 12. Patients who received 2 mg or 3 mg of brexpiprazole had clinically meaningful improvements compared to the placebo group. This approval reflects, at least in part, the significant unmet needs in the management of behavioral symptoms in AD and the related societal pressure. In the case of partial or non-response with antipsychotics, the mood-stabilizing drugs carbamazepine, gabapentin, and valproic acid should be considered. 73 74

Emerging therapeutics for agitation in AD include pimavanserin (discussed above), dextromethorphan/quinidine, and cannabinoids. Dextromethorphan/quinidine is an FDA-approved drug combination for the treatment of pseudobulbar affect (or pathological laughing/crying, involuntary emotional expression disorder) associated with chronic neurological conditions, such as multiple sclerosis and amyotrophic lateral sclerosis. In a randomized phase 2, double-blind, placebo-controlled trial, dextromethorphan/quinidine demonstrated clinically relevant efficacy for agitation and was well tolerated. 75 In a randomized double-blind crossover trial comparing nabilone – a synthetic tetrahydrocannabinol analog – with a placebo, nabilone was effective for agitation but caused sedation and cognitive impairment. 76 A meta-analysis of the literature including natural and synthetic cannabinoids for agitation in AD concluded there may be a signal for a potential benefit of synthetic cannabinoids, but there are concerns with side effects, mainly sedation. 77

In sum, no pharmacological interventions for agitation in AD have therapeutic benefits that clearly outweigh their potential side effects and related safety concerns. 78 Non-pharmacological or psychosocial interventions should be regarded as the mainstay of the treatment of agitation in AD.

## SLEEP DISORDERS

There is a bidirectional relationship between AD and sleep disorders. In older individuals, sleep disorders are risk factors for cognitive impairment and dementia. 79 80 Sleep disorders have also been identified as a predictor of dementia incidence in prodromal phases of AD, such as MCI, 81 with an estimated population attributable risk of AD because of sleep disorders around 15%. 80 Conversely, the prevalence of sleep disorders among individuals with AD ranges from 14% to 69%, with a pooled prevalence estimate of 39% (95% CI 30–47%). 4 The most commonly reported sleep disorders in AD are insomnia, sleep-disordered breathing (SDB), restless legs syndrome, and rapid eye movement (REM) sleep behavior disorder. 81

While sleep disorders may contribute to AD pathophysiology, AD-related pathology contributes to sleep problems. 81 Sleep disorders can exacerbate cognitive symptoms by impairing sleep-dependent memory consolidation processes. 80 Degeneration of neural pathways that regulate sleep-wake cycles and sleep architecture, such as the suprachiasmatic nucleus, can lead to sleep-wake cycle disorders, resulting in fragmented sleep and sleep-wake cycle reversal, with daytime sleepiness and nocturnal agitation. Medications used to treat AD-related symptoms can further contribute to sleep dysfunction. For example, cholinesterase inhibitors can cause vivid dreams and nightmares, while antipsychotic medications can cause sedation. 82

In this context, sleep disorders are highly prevalent among individuals with AD, and their presence worsens disease presentation and may accelerate disease progression. Yet, their management in AD is very limited. Non-pharmacological interventions (e.g., exercise, continuous positive airway pressure (CPAP), bright light therapy) are the first-line approach for sleep problems in AD, as they conciliate a favorable safety profile with reported efficacy. 82 When these interventions do not work, pharmacological strategies can be considered. There is a dearth of evidence of the efficacy of many drugs widely prescribed for sleep disorders in dementia, including benzodiazepine and non-benzodiazepine hypnotics (e.g., zolpidem, eszopiclone). Moreover, the use of these drugs is associated with potentially meaningful side effects, such as risk of falls and cognitive decline. Melatonin is widely used for treating sleep problems in AD, especially due to its positive safety profile and potential to regulate disrupted circadian rhythms. However, the evidence of melatonin efficacy in improving sleep disorders in AD is limited. Although a randomized, placebo-controlled trial showed a significant improvement in subjective sleep measures with melatonin compared to placebo, 83 others found no clinical benefit of melatonin when sleep outcomes were measured objectively (e.g., actigraphy). 84 85 The most recent Cochrane Review on the topic concluded that there is no evidence of the efficacy of melatonin (up to 10 mg) or melatonin receptor agonists (e.g., agomelatine, ramelteon) for sleep problems in AD (including total nocturnal sleep time, mean duration of sleep bouts, sleep efficiency, number of nocturnal awakenings, and sleep latency). 86 There is evidence of potential beneficial effects on sleep outcomes in AD, with a relatively safe profile, for trazodone and orexin antagonists (e.g., suvorexant). 86 There is a clear inconsistency between the current evidence versus the clinical practice to treat sleep disorders in AD. A systematic assessment of the benefits and adverse effects of drugs used to treat sleep problems in AD is urgently needed.

In conclusion, in addition to its core clinical features of cognitive and functional decline, AD is characterized by a series of neuropsychiatric or behavioral symptoms. These symptoms most commonly include apathy, depression, anxiety, agitation, and psychosis, which significantly contribute to the clinical heterogeneity of AD and, thus, to its challenging management. Indeed, they are often debilitating, impacting several outcomes, such as caregiver burden and service utilization. Several non-pharmacological approaches for behavioral symptoms in AD have been proposed and demonstrated to be effective, but there are barriers to their implementation and they may not be enough to control them. Pharmacological treatments have shown modest efficacy, but potentially serious side effects, especially related to antipsychotics. Furthermore, the dynamic nature of these symptoms, which tend to fluctuate in frequency and severity, requires constant reassessment and flexibility of the therapeutic plan. There is a great need to develop safer and more efficacious strategies for the management of behavioral symptoms of AD.
